# Re-evaluating the significance of the dive response during voluntary surface apneas in the bottlenose dolphin, *Tursiops truncatus*

**DOI:** 10.1038/s41598-019-45064-8

**Published:** 2019-06-13

**Authors:** A. Fahlman, S. Miedler, J. Rocho-Levine, A. Jabois, J. Arenarez, L. Marti-Bonmati, D. García-Párraga, F. Cauture

**Affiliations:** 1 Research Group on Biomedical Imaging (GIBI230), Instituto de Investigación Sanitaria la Fe, 46026 Valencia, Spain; 2 Departamento de Investigación, Fundación Oceanogràfic de la Comunidad Valenciana, Gran Vía Marqués del Turia 19, 46005 Valencia, Spain; 3 Veterinary Cardiology, Plaza Mayor 7/10, 46120 Alboraya, Valencia, Spain; 4 Dolphin Quest, Oahu, 5000 Kahala Ave, Honolulu, HI USA; 5 Departamento de Biología, Avanqua-Oceanográfic SL, Gran Vía Marqués del Turia 19, 46005 Valencia, Spain

**Keywords:** Blood flow, Animal physiology

## Abstract

The dive response is well documented for marine mammals, and includes a significant reduction in heart rate (*f*_H_) during submersion as compared while breathing at the surface. In the current study we assessed the influence of the Respiratory Sinus Arrhythmia (RSA) while estimating the resting *f*_H_ while breathing. Using transthoracic echocardiography we measured *f*_H_, and stroke volume (SV) during voluntary surface apneas at rest up to 255 s, and during recovery from apnea in 11 adult bottlenose dolphins (*Tursiops truncatus*, 9 males and 2 females, body mass range: 140–235 kg). The dolphins exhibited a significant post-respiratory tachycardia and increased SV. Therefore, only data after this RSA had stabilized were used for analysis and comparison. The average (±s.d.) *f*_H_, SV, and cardiac output (CO) after spontaneous breaths while resting at the surface were 44 ± 6 beats min^−1^, 179 ± 31 ml, and 7909 ± 1814 l min^−1^, respectively. During the apnea the *f*_H_, SV, and CO decreased proportionally with the breath-hold duration, and after 255 s they, respectively, had decreased by an average of 18%, 1–21%, and 12–37%. During recovery, the *f*_H_, SV, and CO rapidly increased by as much as 117%, 34%, and 190%, respectively. Next, *f*_H_, SV and CO rapidly decreased to resting values between 90–110 s following the surface apnea. These data highlight the necessity to define how the resting *f*_H_ is estimated at the surface, and separating it from the RSA associated with each breath to evaluate the significance of cardiorespiratory matching during diving.

## Introduction

The cardiovascular responses in marine mammals were initially studied during forced dives by Per Scholander^1^ who concluded that during a breath-hold there is a decrease in heart-rate (*f*_H_), and presumably a decrease in cardiac output (CO), and an increase in peripheral resistance (vasoconstriction)^2^. Scholander later coined these cardiovascular responses the “master switch of life” and proposed that this was a reflex that was tightly linked to diving^2^. Following this initial work, numerous studies have measured *f*_H_ during restrained, semi-restrained, and voluntary diving and swimming in a range of breath-hold diving vertebrates^1,3–17^. It has been concluded that there are marked differences in cardiac responses in forced vs. voluntary/natural diving^18^. However, the minimum *f*_H_ during diving in free ranging pinnipeds is similar to those during forced diving experiments, and there was no clear correlation between diving capacity and the level of bradycardia^19^. Based on these differences some have argued that the dive response is not a reflex adjustment^20^, but a more generalized response that is not a specific trait for diving^19^. In addition, some studies have provided evidence that diving mammals possess cognitive control over cardiac function^16,21–23^, which may allow management of gases during diving by altering the ventilation-perfusion matching^24^.

Still, it is universally agreed that diving vertebrates experience a diving related bradycardia while submerged^20,25,26^. However, there is disagreement whether there are changes in stroke volume (SV) during diving^5,9,12,13,15,22,27–30^, and to our knowledge, no study has assessed SV in diving cetaceans. Thus, current estimates of CO in cetaceans, and how they may be modified during diving, are based on estimated values from pinnipeds and terrestrial species. These direct extrapolations could significantly bias our understanding of the eco-physiology of cetaceans and the results of gas dynamics modeling regarding diving performance^31–34^.

In cetaceans, most studies have largely been focused on cardiac frequencies^7,16,17,21,35^, and no published data exists on cardiac contractile responses during forced or voluntary dives. Despite anatomical difficulties obtaining images of the heart, a non-invasive study on cardiac performance using transthoracic echocardiography have previously been published in bottlenose dolphins (*Tursiops truncatus*) before and after high intensity exercise^36^. In cetaceans, the respiratory sinus arrhythmia (RSA) possibly results in a biased average resting *f*_H_ depending on the breathing frequency (*f*_R_). For example, in the bottlenose dolphin the *f*_H_ immediately following a spontaneous breath during rest may be as high as 80–100 beats · min^−1^, but rapidly declines to a stable values around 40–50 beats · min^−1^ in about 8–20 s following the breath^16,17,36,37^. The respiratory phase in cetaceans begins with a rapid expiration, followed by an inspiration and a respiratory pause that can last for up to a minute^38^. Consequently, if the resting *f*_H_ at the surface is estimated over a pre-determined time interval, the RSA may significantly alter the value, and it will vary with the *f*_R_ and the duration of the time interval chosen.

**Figure 1:**
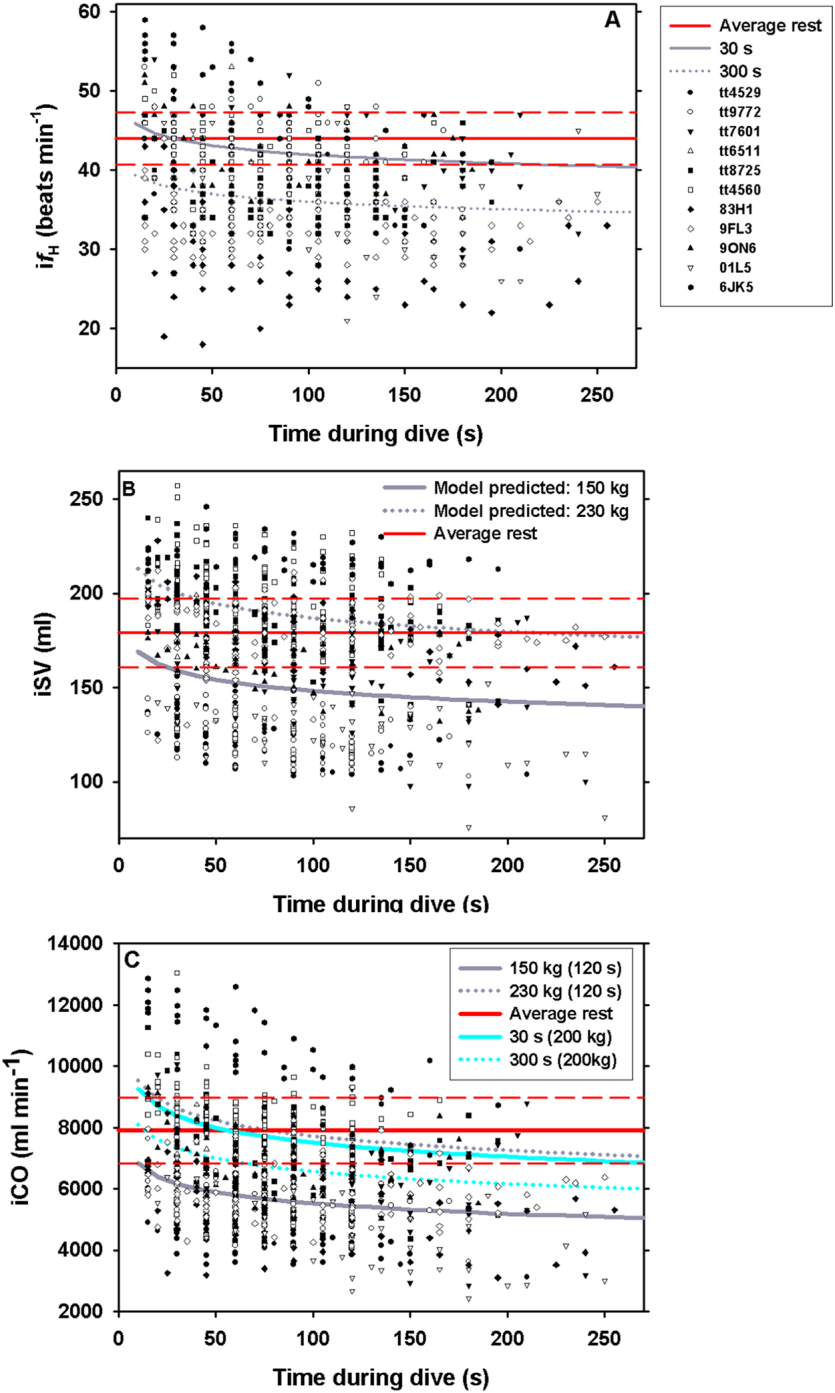
Instantaneous (**A**) heart rate (i*f*_H_, n = 13), (**B**) stroke volume (iSV, n = 11), and (**C)** cardiac output (iCO, n = 11) versus time during dive (s) in bottlenose dolphin. Solid and dotted red lines are the resting average and 95% confidence limits, respectively, while breathing after correcting for respiratory sinus arrhythmia. Gray solid and dotted lines are model predicted regressions for (**A**) maximal dive duration of 30 s or 300 s (**B**) and (**C**) for a 150 kg and 230 kg dolphin (for a 120 s maximal dive duration in (**C**), and cyan solid and dotted lines are model predictions for a maximal dive duration of 30 s or 300 s for a 200 kg animal. Note for *f*_H_, panel A, the model did not warrant inclusion of *M*_b_. The legend in panel A give the symbol for each individual animal in panels A–C.

**Figure 2:**
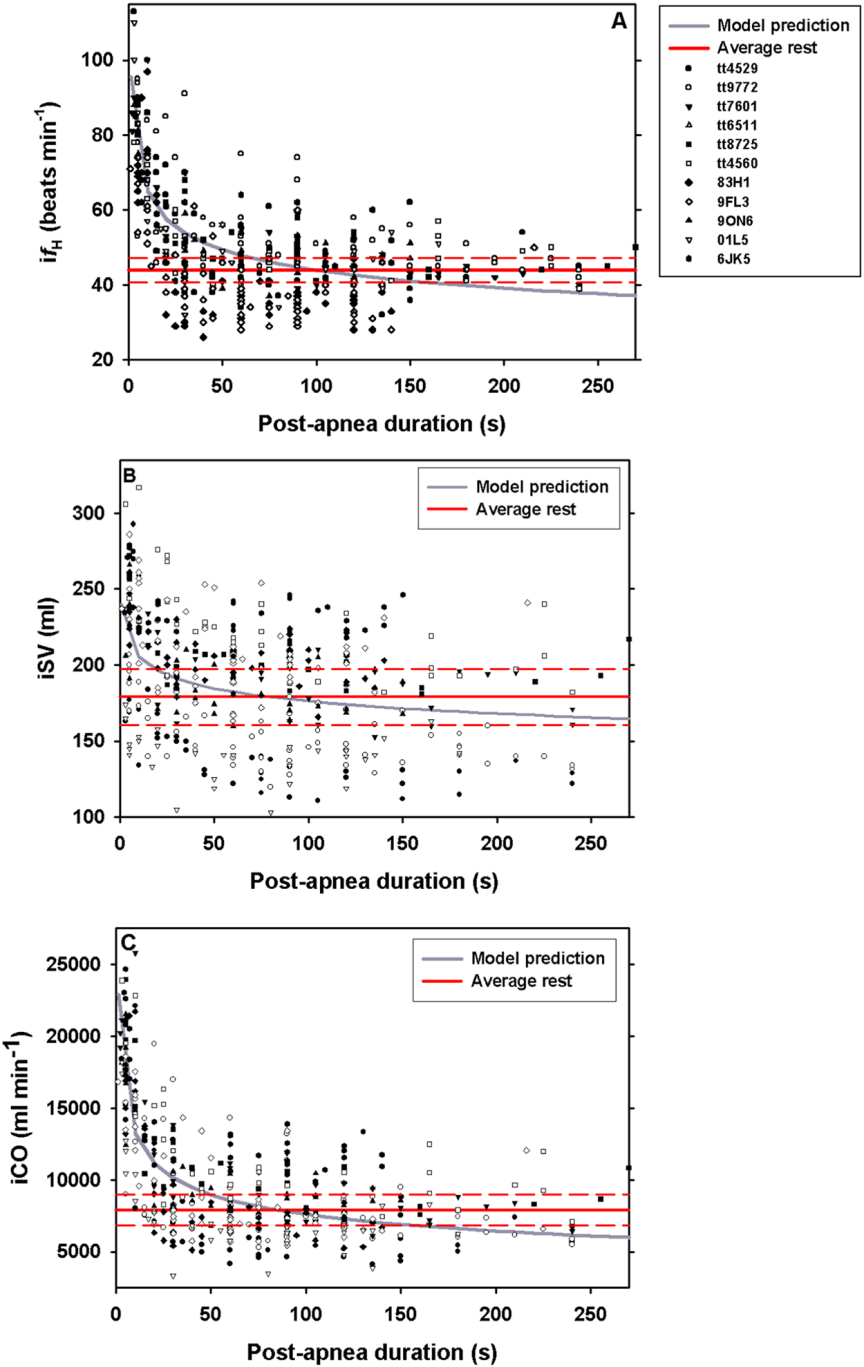
Instantaneous (**A**) heart rate (i*f*_H_, n = 13), (**B**) stroke volume (iSV, n = 11), and (**C**) cardiac output (iCO, n = 11) versus duration following a voluntary surface apnea (s) at rest in bottlenose dolphin. Solid and dotted red lines are the resting average and 95% confidence limits, respectively while breathing after correcting for respiratory sinus arrhythmia. Gray solid line is model predicted regressions. The legend in panel A give the symbol for each individual animal in panels A–C.

Most studies evaluating the cardiac responses during diving and/or exercise in diving mammals have included the *f*_H_ changes caused by the RSA to estimate the resting *f*_H_ at the surface^7,10,16,17,21,35,39^. It has long been recognized that stress significantly affects physiological responses, and researchers have indicated that cardiac responses during forced dives likely affects any extrapolations to normal cardiac function^1,16,40^. Similar to the effect stress have on *f*_H_, the confounding influence of respiration should be separated from the *f*_H_ before conclusions can be made about the cardiovascular changes during a breath-hold^1,20^. Consequently, conclusions from past studies on either forced or freely diving marine mammals may be confounded by the RSA. For this reason, Miedler *et al*.^36^ proposed that the *f*_H_ should be evaluated once the instantaneous *f*_H_ (i*f*_H_) had stabilized, maintaining a more or less constant level during at least 5–7 s, which occurs approximately 8–20 s after the breath (see Figs 1 and 2 in^37^, and see Supplementary Material).

The aim of this study was to provide estimates of cardiac function in the bottlenose dolphin before, during, and following a voluntary surface apnea at rest. These data provide the first voluntary, non-invasive, and semi-continuous measurements of *f*_H_, SV and CO before, during, and after a surface apnea of up to 255 s using transthoracic ultrasound in 11 adult bottlenose dolphins. The species-specific data provided in this study clearly show that the magnitude and importance of the cardiac responses during voluntary diving may have been significantly overestimated in previous studies, and show that the effect of RSA may have biased the results from previous studies. In addition, we show that both *f*_H_ and SV decrease temporally during the apneic period, and show that cardiovascular recovery times are between 70–100 s following voluntary surface apneas at rest. We conclude that the results presented in this study will enhance our ability to more accurately understand the physiological limitations and gas exchange dynamics in cetacean diving physiology, and provide methods to more readily compare results within and between different studies and species.

## Results

### Heart rate, stroke volume and cardiac output during rest

During rest, the average resting instantaneous *f*_H_ (i*f*_H_, 44 ± 6 beats min^−1^, range: 27–67 beats min^−1^, n = 13, P > 0.1), SV (iSV, 179 ± 31 ml, P > 0.3, n = 11), and CO (iCO, 7909 ± 1814 ml min^−1^, P > 0.9) were not significantly affected by *M*_b_ (Table 1). The average mass-specific *f*_H_ (s*f*_H_), SV (sSV), and CO (sCO) were 0.26 ± 0.04 beats min^−1^ kg^−1^, 1.06 ± 0.24 ml kg^−1^, and 47 ± 11 ml min^−1^ kg^−1^, respectively.

**Table 1:** Aquarium site (Site; Oce-Oceanogràfic, DQ-Dolphin Quest), Animal id, sex (F-female, M-male), body mass (*M*_b_, kg), approximate year of birth for wild caught animals or year born for animals born under human care, site born (Origin), aortic valve orifice (AVO) diameter, resting heart rate (*f*_Hrest_), stroke volume (SV_rest_), and cardiac output (CO_rest_).

Site	Animal ID	Sex	*M*_b_ (kg)	Birth date (Mo/Yr)	Origin	AVO (cm)	*f*_Hrest_ (beats min^−1^)	SV_rest_ (ml)	CO_rest_ (ml min^−1^)
Oce	Tt4529	F	159	NA/1989	Wild	3.1	48 ± 11^20^	130 ± 9	6227 ± 1566
Oce	Tt9772	M	164	NA/1992	Wild	3.0	46 ± 5^18^	131 ± 13	6082 ± 1174
Oce	Tt7601	M	182	06/2004	Oce	3.2	39 ± 4^11^	165 ± 21	6501 ± 1143
Oce	Tt6511	M	140	05/2013	Oce	3.2	43 ± 5^22^	178 ± 23	7765 ± 1505
Oce	Tt8725	F	161	08/2003	Barcelona	3.4	45 ± 6^57^	205 ± 19	9232 ± 1779
Oce	Tt4560	M	151	05/2006	Barcelona	3.4	44 ± 5^55^	208 ± 22	9019 ± 1016
Oce	Tt5550	F	146	09/2006	Oce	NA	34 ± 5^12^	NA	NA
DQ	83H1	M	140	03/2008	DQ	3.4	36 ± 5^29^	201 ± 12	7283 ± 1368
DQ	9FL3	M	235	10/1997	Wild	3.2	36 ± 5^29^	194 ± 12	6989 ± 1128
DQ	9ON6	M	184	09/2000	DQ	3.4	49 ± 8^23^	175 ± 11	8630 ± 1756
DQ	01L5	M	155	01/1985	Sea Life Park	3.2	46 ± 6^9^	153 ± 27	6984 ± 1293
DQ	63H4	M	171	03/1991	DQ	NA	52 ± 4^2^	NA	NA
DQ	6JK5	M	207	01/1995	DQ	3.6	55 ± 8^16^	224 ± 12	12282 ± 1713
	Grand Mean	—	169 ± 28	—	—	3.3 ± 0.2	44 ± 6	179 ± 31	7909 ± 1814

*Animals born under human care. The superscript in the *f*_Hrest_ column is number of measurements that were used to determine the average *f*_Hrest_, SV_rest_ and CO_rest_. Each measurement was the average of at least 3 R-R intervals following a breath. NA-Not Available.

### Heart rate, stroke volume and cardiac output during surface apnea

Of the 13 dolphins that participated in the study, the diameter of the aortic valve orifice could be measured in 11 animals (Table 1). These 11 dolphins participated in a total of 173 voluntary surface apneas at rest, ranging in duration from 20 s to 255 s (Table 2). For i*f*_H_, the best model to describe the effect of surface apnea included the maximal duration of the dive, and the time during the dive (Fig. 1A, Table 3). The best models to describe the effect of apnea on iSV included *M*_b_ and apnea duration (Fig. 1B, Table 3). For iCO the best model included *M*_b_, time during the dive, and the maximal duration of each breath-hold (Fig. 1C, Table 3).

**Table 2:** Animal ID, number of breath-hold trials (n), and average (±s.d.) maximal dive duration (MDD, s).

Animal ID	n	MDD (s)
Tt4529	16	115 ± 36
Tt9772	23	120 ± 24
Tt7601	12	165 ± 41
Tt6511	4	78 ± 49
Tt8725	28	110 ± 43
Tt4560	30	98 ± 31
Tt5550	—	—
83H1	15	97 ± 67
9FL3	16	115 ± 70
9ON6	11	113 ± 49
01L5	10	136 ± 70
63H4	—	—
6JK5	8	115 ± 53
Grand Mean	16 ± 8	115 ± 22

**Table 3:** Generalized least square regression results for Log_10_-transformed instantaneous heart rate(Log[i*f*_H_]), instantaneous stroke volume (Log[iSV]), and instantaneous cardiac output (Log[iCO]), against Log_10_-transformed time during dive or recovery (log[time]), maximal dive duration (log[max]), or body mass (log[*M*_b_]). Shown are the parameters (±s.e.) for the best model, the χ^2^, and P-value against nested models and the r^2^_m_/r^2^_c_ are the marginal and conditional r^2^, respectively. SV is in ml, CO in ml · min^−1^, durations in s, and *M*_b_ in kg.

Phase	Dependent variable	β_0_	Log[time]	Log[max]	Log(*M*_b_)	χ^2^	P-ϖαλυε	r^2^_m_/r^2^_c_
Apnea	Log(*f*_H_)	1.80 ± 0.03	−0.039 ± 0.008	−0.067 ± 0.012	—	28.8	<0.01	0.07/0.53
	Log(SV)	1.11 ± 0.26	−0.057 ± 0.005	—	0.54 ± 0.12	20.9	<0.01	0.16/0.82
	Log(CO)	2.35 ± 0.45	−0.091 ± 0.011	−0.059 ± 0.018	0.34 ± 0.20	10.8	<0.01	0.19/0.70
Recovery	Log(*f*_H_)	1.98 ± 0.02	−0.169 ± 0.007	—	—	5.6	<0.05	0.47/0.63
	Log(SV)	2.38 ± 0.03	−0.067 ± 0.004	—	—	202	<0.01	0.12/0.80
	Log(CO)	4.36 ± 0.03	−0.24 ± 0.01	—	—	368	<0.01	0.46/0.68

### Heart rate, stroke volume and cardiac output during recovery

The average (n = 10) i*f*_H_, iSV, and iCO after the 10 first seconds following the first breath from a voluntary surface apnea at rest was 78 ± 8 beats · min^−1^ (t-test compared to pre-dive rest, t-value = 11.1, df = 21 P < 0.01), 225 ± 41 ml (t-test compared to pre-dive rest, t-value = 2.9, df = 19 P < 0.01), and 17612 ± 3047 ml · min^−1^ (t-test compared to pre-dive rest, t-value = 9.0, df = 19 P < 0.01), respectively. For i*f*_H_ (Fig. 2A), iSV (Fig. 2B), and iCO (Fig. 2C, Table 3) the best models to describe the recovery from surface apnea included recovery time. Neither the duration of the surface apnea, nor *M*_b_ warranted inclusion in the model (P > 0.3 for all).

## Discussion

Given the importance to understand the effects of O_2_ delivery during exercise and submersion, determining the cardiovascular responses are important to understand physiological function in cetaceans. The objective with the current study was to evaluate i*f*_H_, iSV and iCO before, during and following voluntary surface apneas at rest to help improve our understanding of cardiorespiratory responses associated with diving. Similar to previous studies, we report a significant RSA^10,16,17,36,37^, which alters how the resting *f*_H_ is evaluated and thereby the magnitude of the apparent dive response. As RSA is known to be affected by both tidal volume (*V*_T_) and *f*_R_ in humans and dolphins^37,41^, we propose that studies that are aimed at evaluating the cardiovascular responses associated with diving and exercise need to evaluate the effect of RSA when estimating the surface *f*_H_ that is made as a comparison. For example, if i*f*_H_ increases to approximately 120 beats · min^−1^ immediately following a dive, what proportion of this increase is merely due to elevated *f*_R_? To answer this, the cardiorespiratory coupling should be determined for each species to accurately determine the effect of RSA (see Fig. 1 in^37^, and Supplemental Material).

The cardiovascular changes associated with diving and exercise while submerged have long fascinated eco-physiologists trying to understand how marine vertebrates manage gases during diving. Most initial studies were performed in a laboratory setting during forced submersions, but the development of bio -logging tools, or use of medical technology have opened up new avenues for research and understanding the cardiorespiratory changes while submerged under natural conditions, or in studies on animals under voluntary control^7,10,17,21,35,37,39,42^. The changes in *f*_H_ associated with diving have been studied intensively in numerous species^26^. Only a few studies have measured CO in live marine mammals, and most of these studies have been made in pinnipeds^5,9,15,22,27–30^. Of these, only three have been performed in unrestrained and/or free swimming animals^9,15,22^. In the sea lion, SV and *f*_H_ were not reported^15^, but in the seal, CO and *f*_H_ increased while SV decreased as the animal was swimming at the surface^9^. A similar increase in CO was observed during exercise in the submerged harbor seal, while SV also increased slightly^9^. We are only aware of one study that has measured SV in cetaceans either at rest or following high intensity exercise^36^. In a study looking at the development of the dive response in bottlenose dolphins, it was reported that there were no differences with age in resting *f*_H_ at the surface, but the *f*_H_ during submersions was reduced to lower values in older animals^7^. In the harbor porpoise, the *f*_H_ before and during diving was altered depending on the anticipated dive duration, suggestive of cognitive control of *f*_H_^21^. In exercising dolphins there was a linear increase in *f*_H_ with increasing metabolic rate^10^. A similar complex relationship was observed in freely swimming and exercising harbor porpoises where the degree of diving bradycardia was altered by the duration and activity level of the submersion^35^. An important detail in the current study is whether the post-breath period that cause variation in *f*_H_ is included in calculating the resting *f*_H_ as this response is pronounced in this taxonomic group and depends on the *f*_R_^37^.

In our past work^36,37^, and based on the result in the current study, we propose that for better interpretation of the magnitude of the cardiac adjustment during a breath-hold, the comparison should be made with surface values that separate the confounding influence of the RSA. There could be different ways to account for this, and one would be to allow for a long enough resting period that assured that the animals *f*_R_ was normal, or at least report the *f*_R_ so that comparisons could be made between studies. However, as that would still involve confounding the autonomic cardiac response with the voluntary process of breathing, an alternative would be to measure the resting heart rate with minimal influence of the RSA. Thus, we propose that it can be done by measuring the i*f*_H_ as we have done in the current study, or continuous ECG measurements that allows the changes in i*f*_H_ to be modeled and the stabilized *f*_H_ predicted^37^. We propose to call this value the RSA-corrected resting *f*_H_, to differentiate this with the resting *f*_H_ reported in most studies.

The average RSA-corrected resting *f*_H_ in the current study was not different from those reported in past studies using the same individual dolphins and the same methodology, or continuously measuring the ECG (*f*_H_ = 41 ± 9 beats min^−1^ n = 13, 2-tailed t-test, P = 0.33, t-value = 1.0, df = 24)^36,37^, but was considerably lower and less variable as compared with resting *f*_H_’s reported in previous studies in the bottlenose dolphin (ranging from 60–105 beats min^−1^)^7,17,39^. The RSA-corrected resting *f*_H_, SV and CO, where estimated to exclude the changes in *f*_H_, SV and CO associated with respiration^36,37^. As both *f*_H_ and SV increase following a breath, our average readings were lower as compared with an average obtained if the *f*_H_ was estimated over the whole inter-breath cycle, which included the RSA (see Fig. 1 in^37^, or Supplemental Material). Conversely, estimating resting *f*_H_ over a determined period of time includes the variation associated with the RSA, and the average will therefore depend on the duration of the measurement and the *f*_R_ within this period.

Thus, the higher *f*_H_ reported in previous studies may be confounded through inclusion of RSA, and without reporting the *f*_R_ a comparison is difficult. Consequently, estimating resting *f*_H_ without accounting for the RSA result in a higher resting value, and if used to assess the dive response it will result in higher values than when using the RSA-corrected resting *f*_H_. For example, in one study in the bottlenose dolphin, the estimated resting *f*_H_ while at the surface was 105 ± 8 beats min^−1^, and decreased to 40 ± 6 beats min^−1^, while submerged at 15 m for an average dive duration of 85 ± 51 s (range 14–160 s)^39^. The RSA-corrected resting *f*_H_ during a voluntary surface apnea up to 160 s in the current study was similar to the resting diving *f*_H_ reported in the past study^39^, and similar to the resting *f*_H_ for spontaneous breathing at the surface after accounting for the RSA (27 to 67 beats min^−1^, see refs ^36,37^). Thus, our results suggest that the changes in *f*_H_ associated with diving, after accounting for RSA, are less pronounced than previously reported, and only become significantly lower than resting surface values during apneas exceeding 2.5–3 min (Fig. 1A). Thus, the results presented here provide an interesting perspective that when accounting for the RSA dolphins do not exhibit the pronounced diving bradycardia associated with the stress during forced dives or those that include the RSA when estimating the resting *f*_H_, except during extended apneas. Whether this is a universal trait in other marine mammals, or how exercise or depth influence these changes remains to be determined once past and future studies evaluate the RSA-corrected resting *f*_H_ in voluntary diving animals. Interestingly, similar results were found in elephant seals where the RSA minimum and *f*_H_ during apnea appeared similar^43^. In addition, the RSA developed with age, and its magnitude correlated with dive duration^43^. Thus, the RSA may be an important physiological index that is more correlated with diving ability in addition to cognitive ability to alter *f*_H_ and SV to accurately match ventilation and perfusion to improve gas exchange^24^.

Both resting sSV, and sCO were significantly higher as compared with a previous study assessing cardiovascular changes following a bout of exercise (sSV = 0.79 ± 0.14 ml · kg^−1^, 2-tailed t-test, P < 0.01, t-value = 3.4, df = 22; sCO = 32 ± 9 ml · min^−1^ · kg^−1^, 2-tailed t-test, P < 0.01, t-value = 3.6, df = 22)^36^. However, both sSV and sCO were considerably lower as compared with those measured at rest in the harbor seal (*Phoca vitulina*, sSv = 1.8–3.1 mL · kg^−1^, sCO = 102–394 mL · min^−1^ · kg^−1^)^9^, and California sea lion (*Zalophus californianus*, sSv = 2.0 mL · kg^−1^, sCO = 150–180 mL · min^−1^ · kg^−1^)^15^ using thermodilution techniques. We believe that these differences between species could in part be explained by the lower *f*_R_, breath duration, and higher flow-rates, and *V*_T_’s in the cetaceans as compared with the pinnipeds^38,44^. Thus, if respiration significantly alters cardiac function (*f*_H_ and SV), it is vital that these variables are compared during periods between breaths to minimize the impact of respiration on cardiac function. By standardizing these measurements to prevent the confounding effect of RSA, it would allow inter- and intra-species comparisons by reducing the variability caused by the RSA.

It has been suggested that the extreme changes in *f*_H_ associated with forced submergence, when an animal does not know or has no control over the duration of the apnea, reflect an animal that prepares for a maximal asphyxic challenge^20^. For this reason, Blix^20^ proposed that studies that assess the dive response should clearly distinguish between forced and voluntary diving. An additional issue that may significantly alter variation in *f*_H_, especially in studies on trained animals, is the suggestion that marine mammals have cognitive control of *f*_H_^20–23^. As repeated measurements were performed for each trial, the time during the dive allowed us to investigate temporal changes during a breath-hold. The maximal dive duration was the total duration of the apnea, which allowed us to assess whether the initial cardiovascular changes were different for a long or short breath-hold, possibly indicating a learning effect or cognitive ability to alter *f*_H_ and SV. Our results showed a greater initial drop in *f*_H_ during longer dives (Fig. 1A), which provide additional support of voluntary control of the diving related changes in *f*_H_^20–23^. Previously, it has been proposed that by fine tuning the alveolar ventilation (${\dot{V}}_{A}$) and lung perfusion, marine vertebrates are able to selectively exchange O_2_ and CO_2_ during diving, while minimizing N_2_ exchange^24^. This mechanism relies in part on voluntary control of pulmonary and systemic perfusion, and the ability to selectively perfuse collapsed regions of the lung^24^. Disruption of such refined control of pulmonary blood flow could have severe consequences in gas management, and may explain how species that are normally able to avoid diving related problems experience gas bubble disease when exposed to stressful situations while diving^32,45–50^. While our study does not provide direct evidence of voluntary control of perfusion, we show indirect evidence that the dolphins alter *f*_H_ and SV depending on the length of the dive, similar to the results in the harbor porpoise^21^. As proposed by Mottishaw *et al*.^19^, the cardiovascular changes during diving may be a complex physiological response altered by a number of factors such as voluntary/anticipatory adjustment, submersion, exercise, stress, and fear. Thus, the cardiovascular responses during diving may be a much more plastic physiological trait rather than a purely autonomic response, or potentially merely an extension of the RSA.

Following a voluntary surface apnea up to 255 s, *f*_H_, SV, and CO increased by 78% (maximal value: 164%), 26% (maximal value: 73%), and 123% (maximal value: 229%), respectively (Figs 1 vs. 2). Similar changes in *f*_H_ at maximal exercise are difficult to assess as they generally do not account for the RSA^10,51^. However, maximal *f*_H_, SV, and CO 10 s following a single bout of high intensity exercise increased by as much as 307%, 294%, and 727%, respectively^36^. Consequently, the cardiovascular changes during recovery from a voluntary surface apnea appear less extreme than measured following a high intensity bout of exercise. There are several possible reasons for the more moderate cardiovascular changes during recovery in the current study. First, the dolphins did not perform high intensity exercise and therefore did not have elevated metabolic rate. The elevated *f*_H_, SV and CO following the breath-hold in the current study was only used to replenish the O_2_ stores and remove any CO_2_ produced caused by the surface apnea. During exercise, the dolphins incur an O_2_ debt that may be exacerbated by the elevated rate of O_2_ consumption. This may require greater cardiovascular recruitment to rapidly repay the greater O_2_ debt following exercise and may extend recovery as compared with voluntary apnea at rest. Increased aerobic work and greater O_2_ debt may explain why i*f*_H_, iSV and iCO took at least 4 min to recover following a high intensity bout of exercise^36^, while in the current study these variables had returned to baseline after approximately 90–110 s (Fig. 2A–C). This agrees with a previous study showing that the O_2_ stores had recovered after approximately 1.2 min following a surface apnea of up to 5 min^52^. Consequently, for an actively diving dolphin we would expect the recovery *f*_H_, SV and CO to reach higher values and/or increase for a longer duration as higher activity would increase the O_2_ debt. In grey seals, the surface interval increased with dive duration for short dives (<7 min) but not for long dives^53^. In extreme divers, such as Weddell and elephant seals there does not appear to be a clear relationship between dive duration, even those exceeding the aerobic dive limit, and surface interval duration^54,55^. One reason could be that seals partially recover while diving^55^. However, the surface *f*_H_ increased with dive duration in the elephant seal^54^, but there was no clear relationship in the grey seal^53^. Similarly, the recovery *f*_H_ during single dives correlated with dive duration and activity in the Steller sea lion^14^. Thus, these data provide evidence for excellent control of cardiovascular function to rapidly and efficiently manage metabolic gases.

In summary, this study provides the first non-invasive measurements of *f*_H_ and SV before, during, and after voluntary surface apneas at rest in cetaceans. To account for the RSA, we measured the RSA-corrected resting i*f*_H_ and iSV only during inter-breath periods when *f*_H_ and SV had stabilized following the breath. We therefore report lower average *f*_H_ while resting at the surface, and propose that these results have lower variability and provide improved comparable values across individuals or species with varying respiratory effort. Further work should be done to define the significance of the RSA, its role in managing gas exchange, and the temporal changes to allow the RSA-corrected resting *f*_H_ and SV to be defined within and between species. The i*f*_H_, iSV and iCO decreased slightly with breath-hold duration as compared with the RSA-corrected resting i*f*_H_. However, compared to past studies, the level of the diving bradycardia was considerably reduced. Following the apnea, the i*f*_H_, iSV and iCO immediately increased as much as 164%, 73%, and 229%, respectively even during the stabilization period, but rapidly returned to resting levels within 50–60 s after several respirations. Based on our results, we propose that future studies that assess the diving related changes in cardiac function have to do similar correction to avoid the confounding effect of RSA, and past studies may have to be re-evaluated to tease apart the contradictory effects of submergence and exercise. Our data show that the initial diving i*f*_H_ is lower during longer breath-hold, which provides additional support that cetaceans have partial cognitive control over their diving bradycardia. This, provides additional support for the hypothesis that cetaceans can voluntarily alter gas exchange during diving.

## Material and Methods

### Animals

The study protocols were approved by the Animal Care and Welfare Committee of the Oceanogràfic Foundation (OCE-17-16 and amendment OCE-29-18), and all experiments were performed in accordance with relevant guidelines and regulations. Ultrasound continuous flow Doppler was used to measure *f*_H_ and SV at the level of the left ventricular outflow tract (at the level of the aortic valve orifice), during and following a surface apnea (Table 1) from 10 adult male and 3 adult female Atlantic bottlenose dolphins, 4 to over 33 years old, housed at the Oceanogràfic-Valencia and Dolphin Quest-Oahu, between 2016 and 2018 (Table 1).

The animal ID, body mass, and age (known or estimated) at the time of the study are summarized in Table 1.

### Experimental trials

All experiments were performed using operant conditioning. Participation by the dolphins was voluntary, and the animals were not restrained and could refuse to participate or withdraw at any point during the experimental trial. Each experiment (trial) consisted of an animal staying stationary in the water in left lateral recumbence with the blow-hole out of the water, allowing ultrasound probe placement to find the left ventricle. To evaluate the effect of a surface apnea, the animals were conditioned to hold their breaths voluntarily as much as possible up to a maximum of 255 s (Table 2). The dolphins were asked by the trainer to turn on their side with the blowhole submerged until they decided to end the breath-hold. The ultrasound examination continued throughout the apnea and recovery period to assess the cardiovascular changes during a breath-hold and recovery. For measurements of *f*_H_, and SV during apnea and recovery, the breath-hold and recovery durations were recorded. Out of the 13 animals, we were only able to measure the aortic diameter in 11 and SV and CO were therefore only estimated in 11 dolphins (Table 1). For that reason, only 11 animals participated in the voluntary breath-hold (Table 2).

### Ultrasound data acquisition

The ultrasound machine (Vivid-I, General Electric) with a 1–3 MHz phased array probe was used to obtain left ventricular i*f*_H_ and iSV, as previously detailed^36^. CO was estimated as: iCO = i*f*_H _× iSV. The i*f*_H_ was estimated from the flow traces, and the iSV calculated from the surface area of the aortic valve orifice multiplied by the velocity time integral of the systolic blood flow along the left ventricular outflow tract. The cross-sectional area of the aortic valve was calculated from the aortic valve orifice diameter as: surface area = radius^2^ × π. In order to obtain left ventricular systolic blood flow, the “apical” long axis view of the left ventricular outflow tract and the aortic root was used to place the continuous flow Doppler parallel to the blood flow in the left ventricle and through the middle of the aortic valve orifice. We measured the aortic valve orifice diameter at the level of valve insertion at the different cardiac phases to confirm that its diameter was circular and constant for all flow-rates.

Our pas studies and initial assessments indicated significant changes in both i*f*_H_ and iSV following respiration^36,37^. Because of this significant respiratory sinus arrhythmia (RSA), at least 3 repeated measurements of *f*_H_ and ventricular flow were made for each measurement and the average used as an estimate of the *f*_H_ following a breath. To obtain resting data that were not affected by the RSA, we only analysed *f*_H_ at least 12–20 s following a spontaneous breath and after the i*f*_H_ had stabilized for 5–7 s following the breath (see Supplementary Material)^36,37^. At this point, the i*f*_H_ and iSV were averaged for at least 3 R-R intervals for each measurement. These were then averaged for each individual. The ultrasound data were saved either as 8–16 s movies or 2-dimensional images and later analysed using manufacturer specific software (See S1 in Supplementary Material).

The duration to locate the aortic valve orifice differed between animals based on the size of the cardiac acoustic window, animal movement, and whether the animal was performing the surface apnea or breathing spontaneously. We standardized the recovery phase to begin immediately following the first breath, and cardiovascular measurements began as early as 10 s following this breath when the i*f*_H_ again stabilized following the RSA.

### Data assessment and statistical analysis

We separated the analysis into one of three conditions: pre-apnea, apnea, post-apnea. The relationship between a dependent variable (CO, SV and *f*_H_) and experimental covariates; body mass (*M*_b_), time during dive, the total dive duration for a given dive (max duration), or time since end of apnea (recovery time), was analyzed using linear-mixed effects models (lme, R: A Language and Environment for Statistical Computing, R Foundation for Statistical Computing, version 3.3.3, 2016). We log_10_-transformed *f*_H_, SV, CO, time during dive, max dive duration, recovery time and *M*_b_ to generate linear functions that could be used with the lme function in R. The individual animal was treated as a random effect, which accounted for the correlation between repeated measurements on the same individual^56^. Initially, a univariate analysis on each independent variable was performed; only those variables with a P-value < 0.10 (Wald’s tests) were considered in a multivariate analysis. Best models of remaining variables were chosen by the log-likelihood (LL) ratio test, and the Akaike information criterion (AIC) and significant parameters assessed by the t-value between the estimate and its standard error. Acceptance of significance was set to the P < 0.05 level, while 0.05 < P < 0.1 was considered a trend. Data are presented as the mean ± standard deviation (s.d.), unless otherwise stated.

## Supplementary information

Supplementary Information

## Data Availability

The data used in this study are freely available at the following link: osf.io/wdfmz.
