# Supplementary material for: Re-evaluating the significance of the dive response during voluntary surface apneas in the bottlenose dolphin, Tursiops truncatus
Source: Sci Rep. 2019 Jun 13;9:8613. doi: 10.1038/s41598-019-45064-8 (PMC6565721; doi:10.1038/s41598-019-45064-8)
Supplement: Supplementary file 1 — Supplementary Information [file 41598_2019_45064_MOESM1_ESM.docx]

Re-evaluating the significance of the dive response during voluntary surface apneas in the bottlenose dolphin, *Tursiops truncatus*

^1,2*^Fahlman, A.,^2,3^Miedler, S., ^4^Rocho-Levine, J., ^5^Jabois, A., ^5^Arenarez, J., ^1^Marti-Bonmati, L., ^2,5^García-Párraga, D., ^2^Cauture, F.

**Supplementary material:**

**S1.** Transthoracic ultrasound recording (2-dimensional images) for a bottlenose dolphin during rest at the surface. Data showing heart rate and aortic flow during rest immediately following a breath (left), or during the inter-breath pause (right). The RSA-corrected resting instantaneous heart rate (i*f*_H_) was the average i*f*_H_ from around time -9 s in the right panel for 5-7 s.
